# Exosome autoantibody biomarkers for detection of lung cancer

**DOI:** 10.1186/s40779-024-00575-y

**Published:** 2024-11-18

**Authors:** Win Lwin Thuya, Janique Michelle Peyper, Tan Ti Myen, Nur Diana Anuar, Arif Anwar, Ranga Gudimella, Nurul Huda Rutt, Nurul Shielawati Mohamed Rosli, Noorul Hidayah Badri, Teh Norleila Abdul Rahman, Raja Nurashirin, Gautam Sethi, John Kit Chung Tam, Andrea Li-Ann Wong, Ross Soo, Jonathan M. Blackburn, Lingzhi Wang, Boon Cher Goh

**Affiliations:** 1 Cancer Science Institute of Singapore, National University of Singapore, Singapore, 117599 Singapore; 2 Sengenics Corporation, Jalan Kerinchi, Bangsar South, 59200 Kuala Lumpur, Malaysia; 3 University of Cape Town, Cape Town, 7700 South Africa; 4 Department of Pharmacology, Yong Loo Lin School of Medicine, National University of Singapore, Singapore, 117600 Singapore; 5 NUS Centre for Cancer Research (N2CR), Yong Loo Lin School of Medicine, National University of Singapore, Singapore, 117599 Singapore; 6 Department of Surgery, Yong Loo Lin School of Medicine, National University of Singapore, 15 1E Kent Ridge Road, Singapore, 119228 Singapore; 7 Department of Haematology-Oncology, National University Cancer Institute, National 17 University Health System, Singapore, 119228 Singapore

**Keywords:** Non-small cell lung cancer, Tumour-derived exosomes, Autoantibodies, Diagnosis, Prognosis

Dear Editor,

Lung cancer remains the leading cause of cancer-related deaths [1], with non-small cell lung cancer (NSCLC) accounting for 85% of cases. Prognosis largely depends on the stage at diagnosis [2]; stage Ia patients have a 5-year survival rate of about 70%, compared to under 20% overall [3]. This highlights the need for effective screening. Currently, low-dose computed tomography (LDCT) is the preferred screening method for high-risk individuals, but it is expensive and has a high false-positive rate, often requiring invasive biopsies. Singapore’s 2010 Cancer Screening guidelines do not recommend chest radiography, sputum cytology, or CT for lung cancer screening [4].

Invasive tissue biopsies pose significant risks and are often impractical when tumours are very small in the early-stages of lung cancer. This underlines the urgent need for simpler, minimally invasive screening methods for early detection. Here we have focused on developing biomarkers from peripheral biofluid-borne extracellular vesicles (EVs). In particular, exosomes, also called small EVs (sEVs), are promising sources of diagnostic and predictive clinical biomarkers [5], since they carry materials that reflect the content of their originating cells, including tumour cells. Notably, cancer cells often release more sEVs due to increased proliferation and inflammatory states, which leads to higher concentrations of tumour-associated antigens (TAAs) in sEVs from cancer patients. Furthermore, tumour-derived sEVs can interact with recipient cells to facilitate processes like immune response modulation and tumour metastasis. We therefore hypothesized that tumour-derived sEVs may be enriched in anti-TAA autoantibodies relative to plasma and that they might thus represent a substantially less invasive source in which to discover and validate novel autoantibody biomarkers of NSCLC.

To test this hypothesis, a clinical study was conducted in National University Hospital, Singapore (Protocol #NS02/04/09) in accordance with the principles of the Declaration of Helsinki. The discovery cohort comprised 109 NSCLC cases (31 early-stage and 78 late-stage) and 100 age-matched healthy controls, while the validation cohort comprised 156 NSCLC cases (30 early-stage and 126 late-stage) and 83 age-matched healthy controls (Additional file 1: Table S1). In discovery phase, exosomes purified from plasma were assayed on iOme v3 microarrays (Sengenics) comprising ca. 1600 purified, full-length, natively-folded human protein antigens. Nineteen biomarker candidates were selected based on penetrance-based fold change (pFC) analysis (Additional file 1: Fig. S1). A custom NSCLC antigen microarray (CAg Plex Co-screen Mini-array) was fabricated comprising just these 19 candidates (Additional file 1: Table S2) and used for biomarker validation in a large independent cohort, comparing the quantitative autoantibody profiles of NSCLC patients to those of healthy controls. In the validation cohort, all (19/19; 100.0%) biomarker candidates achieved pFC ≥ 2 and penetrance frequency ≥ 10% in distinguishing NSCLC cases from controls, with anti-*XAGE1D* exhibiting elevation in the highest proportion (23.7%) of cases; most (89.5%, 17/19) of biomarker candidates achieved pFC ≥ 2 and penetrance frequency ≥ 10% in distinguishing early-stage NSCLC cases from controls, with anti-*RAD23B* exhibiting elevation in the highest proportion (26.7%) of cases (Additional file 1: Table S3). The resulting non-redundant list of candidate discriminators first underwent functional enrichment analysis, followed by receiver operating characteristics (ROC) analysis to evaluate their discriminatory performance in the validation dataset. Finally, recursive feature elimination was applied to identify the candidate discriminator panel best able to distinguish between NSCLC cases and healthy controls. Through this, we identified 4 top autoantibody biomarker panels based on ROC curves (Fig. 1). Among them, a seven-marker panel (anti-*XAGE1D,* -*LRRFIP2*, -*MAGEA10*, -*GAGE2C*, -*STAT1*, -*ZNRD1*, and -*RAD23B*) with an area under the curve (AUC) of 0.818, a sensitivity of 0.753, and a specificity of 0.721 that can differentiate between NSCLC patients and healthy controls, even at early stages of tumourigenesis. Our findings suggest that a panel of exosome-associated autoantibodies can provide a reliable method for early detection of NSCLC, potentially improving upon current screening methods like LDCT. The standout individual biomarker capable of distinguishing NSCLC cases (including early-stage) from controls across both the discovery and validation datasets was anti-*XAGE1D*, which shows potential for detecting lung cancer in a clinical setting. The seven-marker panel could be used in conjunction with risk factors, clinical history, and other laboratory markers to enhance diagnostic accuracy. Additionally, these autoantibodies seemed to reflect various NSCLC molecular subtypes and 2 distinct early-stage NSCLC clusters. Separate per-class unsupervised clustering, based on per-marker candidate fold-change, reveals 2 distinct early-stage NSCLC clusters, 3 distinct late-stage NSCLC clusters (highlighted by green rectangles), and 2 distinct healthy control clusters, as shown in Additional file 1: Fig. S2. Furthermore, identifying humorally “hot” and “cold” subgroups of NSCLC could aid in guiding treatment and prognostication. A systematic review on tumour-associated autoantibody (TAAb) biomarkers in lung cancer diagnosis reported an average TAAb panel AUC of 0.82, highlighting the effectiveness of TAAb panels over individual TAAbs [3]. Our study’s biomarker panel demonstrates similarly high diagnostic potential, reinforcing the utility of multiplexed diagnostic platforms.

**Fig. 1:**
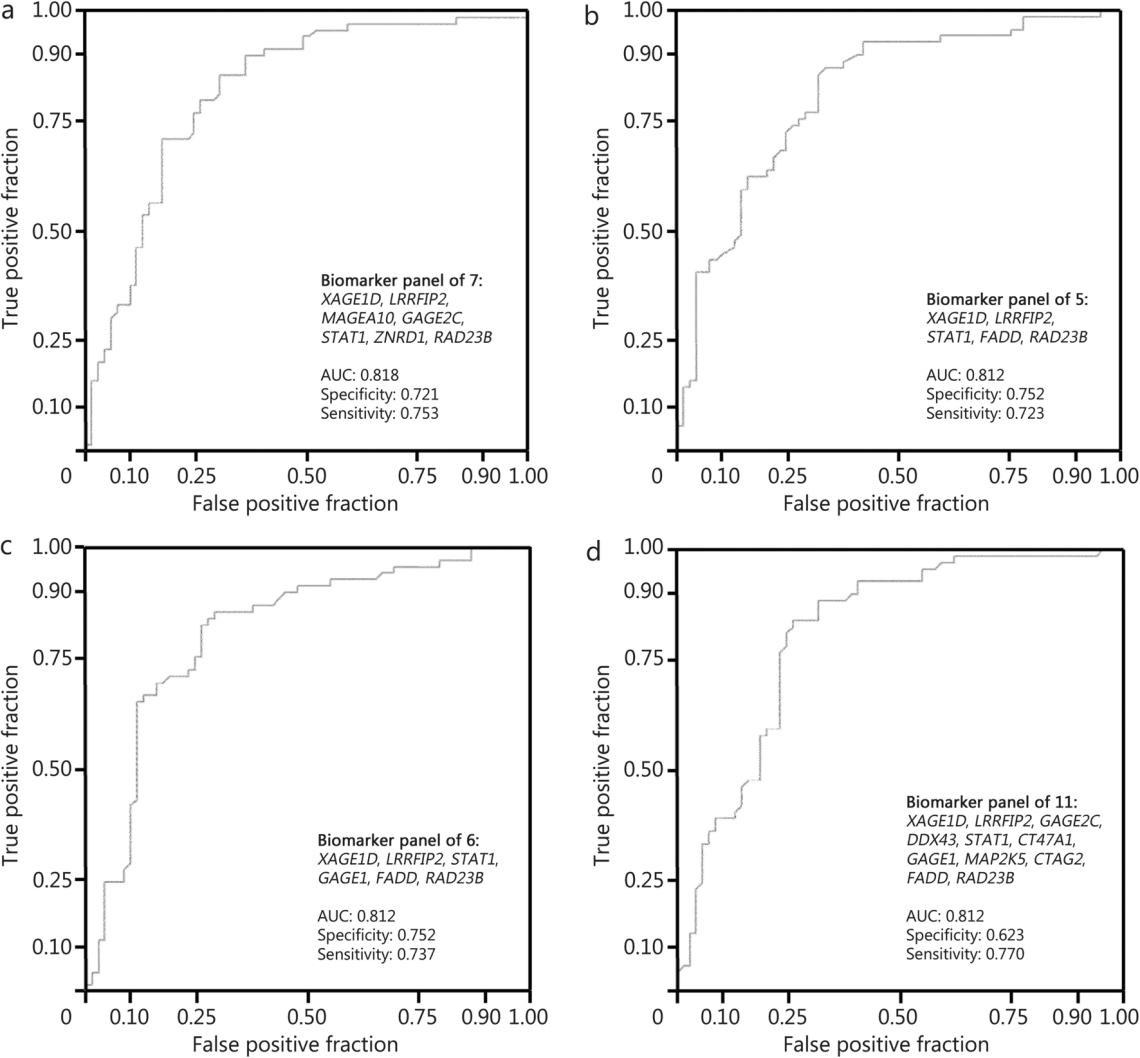
Top 4 autoantibody biomarker panel receiver operating characteristics (ROC) curves. **a** AUC curve of 7 biomarker panel; **b** AUC curve of 5 biomarker panel; **c** AUC curve of 6 biomarker panel; **d** AUC curve of 11 biomarker panel. AUC area under the curve, XAGE1D X antigen family, member 1D, LRRFIP2 LRR binding FLII interacting protein 2, MAGEA10 MAGE family member A10, GAGE2C G antigen 2C, STAT1 signal transducer and activator of transcription 1, ZNRD1 RNA polymerase I subunit H, RAD23B RAD23 homolog B, FADD Fas associated via death domain, GAGE1 G antigen 1, DDX43 DEAD-box helicase 43, CT47A1 cancer/testis antigen family 47 member A1, MAP2K5 mitogen-activated protein kinase kinase 5, CTAG2 cancer/testis antigen 2

In conclusion, the development of non-invasive biomarkers like exosome-associated autoantibodies offers a promising advancement for early NSCLC detection and treatment. When combined with LDCT, this approach may significantly improve specificity in identifying early-stage lung cancer, reducing both patient anxiety and costs associated with false positives and unnecessary invasive procedures. EVs, protected by lipid bilayers that safeguard their cargo (e.g., autoantibodies), provide stability and reflect tumour heterogeneity, making them valuable in diagnosis, prognosis, and treatment prediction. However, challenges remain, such as the lack of standardized protocols for EV isolation and characterization. Further technological advances and validation in larger cohorts are necessary to integrate these biomarkers into clinical practice and revolutionize lung cancer screening with a more accurate, cost-effective, and minimally invasive process.

## Supplementary Information

**Additional file 1: Table S1** The patient sociodemographic and clinicopathological characteristics.** Table S2** Targets selected for inclusion on custom array.** Table S3** Custom array pFC analysis results.** Fig. S1** Per-specificity boxplots of candidate autoantibody biomarker signal intensities, with overlaid grouped scatterplots.** Fig. S2** Fold-change heatmap demonstrating autoantibody-based per-class sub-clusters.

## Data Availability

The datasets used and/or analysed during the current study are available from the corresponding author on reasonable request.

## Abbreviations

NSCLC: Non-small cell lung cancer
LDCT: Low-dose computed tomography
pFC: Penetrance-based fold change
TAA: Tumour-associated antigens
TAAb: Tumour-associated autoantibody
EVs: Extracellular vesicles
sEVs: Small EVs
