# Supplementary material for: Exosome autoantibody biomarkers for detection of lung cancer
Source: Mil Med Res. 2024 Nov 18;11:72. doi: 10.1186/s40779-024-00575-y (PMC11571996; doi:10.1186/s40779-024-00575-y)
Supplement: Supplementary file 1 — Additional file 1: Table S1 The patient sociodemographic and clinicopathological characteristics. Table S2 Targets selected for inclusion on custom array. Table S3 Custom array pFC analysis results. Fig. S1 Per-specificity boxplots of candidate autoantibody biomarker signal intensities, with overlaid grouped scatterplots. Fig. S2 Fold-change heatmap demonstrating autoantibody-based per-class sub-clusters. [file 40779_2024_575_MOESM1_ESM.pdf]

Table S1 The patient sociodemographic and clinicopathological characteristics

Characteristic	Discovery cohort (*n* = 209)		Validation cohort (*n* = 239)	
	**NSCLC patients (*n* = 109)**	**Healthy controls (*n* = 100)**	**NSCLC patients (*n* = 156)**	**Healthy controls (*n* = 83)**
Age (years, mean ± SD)	63 ± 10	59 ± 5	61 ± 11	57 ± 6
Gender [*n*(%)]				
Male	63(57.8)	40(40.0)	99(63.5)	41(49.4)
Female	46(42.2)	60(60.0)	57(36.5)	42(56.6)
Ethnicity [*n*(%)]				
Chinese	78(71.6)	94(94.0)	101(64.7)	69(83.1)
Malay	14(12.8)	2(2.0)	35(22.4)	4(4.8)
Indian	4(3.7)	2(2.0)	6(3.8)	7(8.4)
Others	13(11.9)	2(2.0)	14(9.0)	3(3.6)
Smoking status [*n*(%)]				
Smoker	50(45.9)	3(3.0)	65(41.7)	0
Non-smoker	52(47.7)	97(97.0)	81(51.9)	81(81.0)
Ex-smoker	7(6.4)	0	10(6.4)	2(2.0)
NSCLC stage [*n*(%)]				
Early (< IIIa)	26(23.9)	NA	19(12.2)	NA
Late (≥ IIIa)	83(76.1)	NA	137(87.8)	NA
EGFR gain of function mutation status [*n*(%)]				
Positive	32(29.4)	NA	NA	NA
Negative	30(27.5)	NA	NA	NA
Unknown	47(43.1)	NA	NA	NA

NSCLC non-small cell lung cancer, *EGFR* epidermal growth factor receptor, *NA* not available

Table S2 Targets selected for inclusion on custom array

Target autoantigen	Identified by oFC (No. of comparisons)	Identified by pFC (No. of comparisons)	ROC AUC	Known CT antigen?	No. of criteria met
XAGE1D	Y (3)	Y (4)	0.72	Y	4
STAT1	Y (1)	Y (2)	0.76	N	2
CTAG1A	Y (2)	Y (2)	Not ID'd	Y	2
CTAG2	Y (1)	Y (2)	Not ID'd	Y	2
CT47A1	Y (1)	Y (1)	0.72	Y	3
DDX53	Y (1)	Y (1)	0.70	Y	3
MAGEA4	N	Y (3)	0.73	Y	3
MAGEA10	N	Y (3)	0.72	Y	3
TPM1	N	Y (3)	0.78	N	2
LRRFIP2	N	Y (3)	0.71	N	2
FADD	N	Y (2)	0.71	N	2
RAD23B	N	Y (3)	0.67	N	2
ZNRD1	N	Y (3)	0.66	N	2
MAP2K5	N	Y (3)	0.68	N	2
PTPN20A	N	Y (3)	0.65	N	2
IGF2BP3	N	Y (2)	0.68	Y	2
DDX43	N	Y (2)	Not ID'd	Y	2
GAGE1	N	Y (2)	Not ID'd	Y	2
GAGE2C	N	Y (2)	Not ID'd	Y	2

Not ID'd the antibody targeting the antigen was not identified as a discriminator candidate in the late- vs. early-stage non-small cell lung cancer (NSCLC) comparison, *pFC* penetrance-based fold change, *oFC* overall fold change , *ROC* receiver operator characteristic, *AUC* area under the curve, *CT* computed tomography, *Y* yes, *N* no, *XAGE1D* X antigen family, member 1D, *STAT1* signal transducer and activator of transcription 1, *CTAG1A* cancer/testis antigen 1A, *CTAG2* cancer/testis antigen 2, *CT47A1* cancer/testis antigen family 47 member A1, *DDX53* DEAD-box helicase 53, *MAGEA4* MAGE family member A4, *MAGEA10* MAGE family member A10, *TPM1* tropomyosin 1, *LRRFIP2* LRR binding FLII interacting protein 2, *FADD* Fas associated via death domain, *RAD23B* RAD23 homolog B, nucleotide excision repair protein, *ZNRD1* RNA polymerase i subunit H, *MAP2K5* mitogen-activated protein kinase kinase 5, *PTPN20A* protein tyrosine phosphatase non-receptor type 20, *IGF2BP3* insulin like growth factor 2 MRNA binding protein 3, *DDX43* DEAD-box helicase 43, *GAGE1* G antigen 1, *GAGE2C* G antigen 2C

Table S3 Custom array pFC analysis results

Comparison	Candidate biomarker target	pFC	Penetrance frequency (%)
NSCLC vs. controls	*XAGE1D*	4.57	23.7
	CTAG2	3.77	18.6
	CTAG1A	7.65	18.0
	RAD23B	2.72	16.7
	STAT1	2.64	16.0
	MAGEA4	5.58	15.4
	MAGEA10	2.66	15.4
	LRRFIP2	2.64	15.4
	IGF2BP3	3.05	14.7
	ZNRD1	2.84	14.7
	PTPN20A	2.61	14.7
	DDX53	12.64	14.1
	CT47A1	2.66	14.1
	MAP2K5	2.75	13.5
	GAGE1	2.66	13.5
	DDX43	2.61	13.5
	GAGE2C	2.80	11.5
	FADD	2.73	11.5
	TPM1	2.82	10.3
Early-stage NSCLC vs. controls	*RAD23B*	2.36	26.7
	GAGE1	2.37	16.7
	DDX43	2.33	16.7
	XAGE1D	6.61	13.3
	LRRFIP2	2.60	13.3
	MAGEA4	2.59	13.3
	MAGEA10	2.39	13.3
	GAGE2C	2.36	13.3
	CT47A1	2.32	13.3
	PTPN20A	2.31	13.3
	MAP2K5	2.29	13.3
	STAT1	2.29	13.3
	ZNRD1	2.28	13.3
	TPM1	2.85	10.0

	CTAG1A	2.47	10.0
	IGF2BP3	2.47	10.0
	CTAG2	2.38	10.0
Late-stage NSCLC vs. controls	*XAGE1D*	4.32	26.2
	CTAG2	3.93	20.6
	CTAG1A	8.28	19.8
	STAT1	2.70	16.7
	DDX53	13.67	15.9
	MAGEA4	6.17	15.9
	IGF2BP3	3.13	15.9
	MAGEA10	2.72	15.9
	LRRFIP2	2.65	15.9
	ZNRD1	2.96	15.1
	PTPN20A	2.67	15.1
	RAD23B	2.88	14.3
	CT47A1	2.74	14.3
	MAP2K5	2.86	13.5
	FADD	2.79	12.7
	GAGE1	2.75	12.7
	DDX43	2.70	12.7
	GAGE2C	2.93	11.1
	Anti-*TPM1*	2.81	10.3
Early- vs. late-stage NSCLC	*DDX53*	14.61	12.7
	CTAG1A	9.87	11.1
	XAGE1D	3.75	10.3

NSCLC non-small cell lung cancer, *pFC* penetrance-based fold change, *XAGE1D* X antigen family, member 1D, *STAT1* signal transducer and activator of transcription 1, *CTAG1A* cancer/testis antigen 1A, *CTAG2* cancer/testis antigen 2, *CT47A1* cancer/testis antigen family 47 member A1, *DDX53* DEAD-box helicase 53, *MAGEA4* MAGE family member A4, *MAGEA10* MAGE family member A10, *TPM1* tropomyosin 1, *LRRFIP2* LRR binding FLII interacting protein 2, *FADD* Fas associated via death domain, *RAD23B* RAD23 homolog B, nucleotide excision repair protein, *ZNRD1* RNA polymerase i subunit H, *MAP2K5* mitogen-activated protein kinase kinase 5, *PTPN20A* protein tyrosine phosphatase non-receptor type 20, *IGF2BP3* insulin like growth factor 2 MRNA binding protein 3, *DDX43* DEAD-box helicase 43, *GAGE1* G antigen 1, *GAGE2C* G antigen 2C

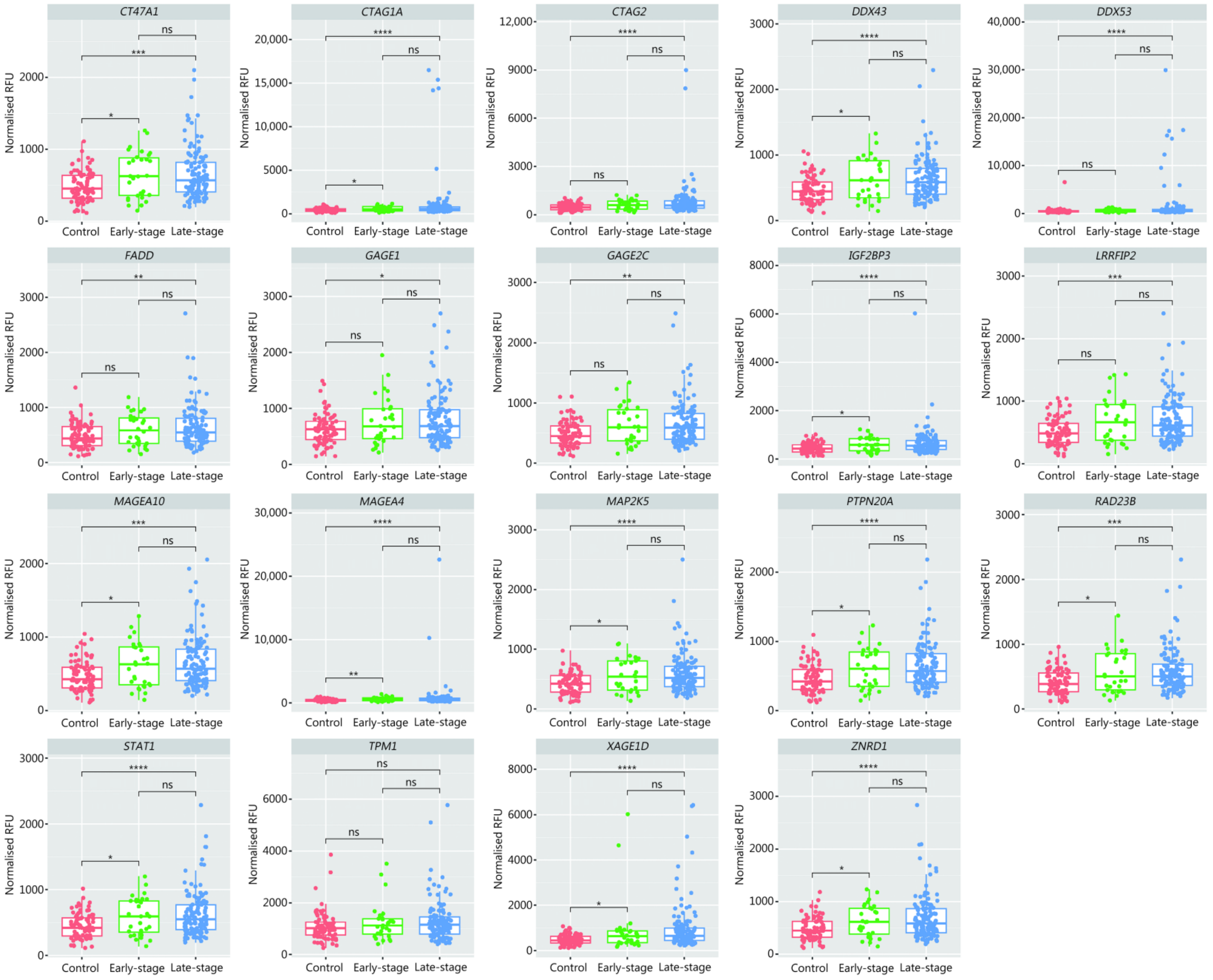

CT47A1
CTAG1A
CTAG2
DDX43
DDX53
FADD
GAGE1
GAGE2C
IGF2BP3
LRRFIP2
MAGEA10
MAGEA4
MAP2K5
PTPN20A
RAD23B
STAT1
TPM1
XAGE1D
ZNRD1
Normalised RFU
Control
Early-stage
Late-stage
ns
*
**
***
****

Fig. S1 Per-specificity boxplots of candidate autoantibody biomarker signal intensities, with overlaid grouped scatterplots. $^{*}P<0.05$, $^{**}P<0.01$, $^{***}P<0.001$, $^{****}P<0.0001$, ns not significant. RFU relative fluorescence units, CT47A1 cancer/testis antigen family 47 member A1, CTAG1A cancer/testis antigen 1A, CTAG2 cancer/testis antigen 2, DDX43 DEAD-box helicase 43, DDX53 DEAD-box helicase 53, FADD Fas associated via death domain, GAGE1 G antigen 1, GAGE2C G antigen 2C, IGF2BP3 insulin like growth factor 2 MRNA binding protein 3, LRRFIP2 LRR binding FLII interacting protein 2, MAGEA10 MAGE family member A10, MAGEA4 MAGE family member A4, MAP2K5 mitogen-activated protein kinase kinase 5, PTPN20A protein tyrosine phosphatase non-receptor type 20, RAD23B RAD23 homolog B, nucleotide excision repair protein, STAT1 signal transducer and activator of transcription 1, TPM1 tropomyosin 1, XAGE1D X antigen family, member 1D, ZNRD1 RNA polymerase i subunit H

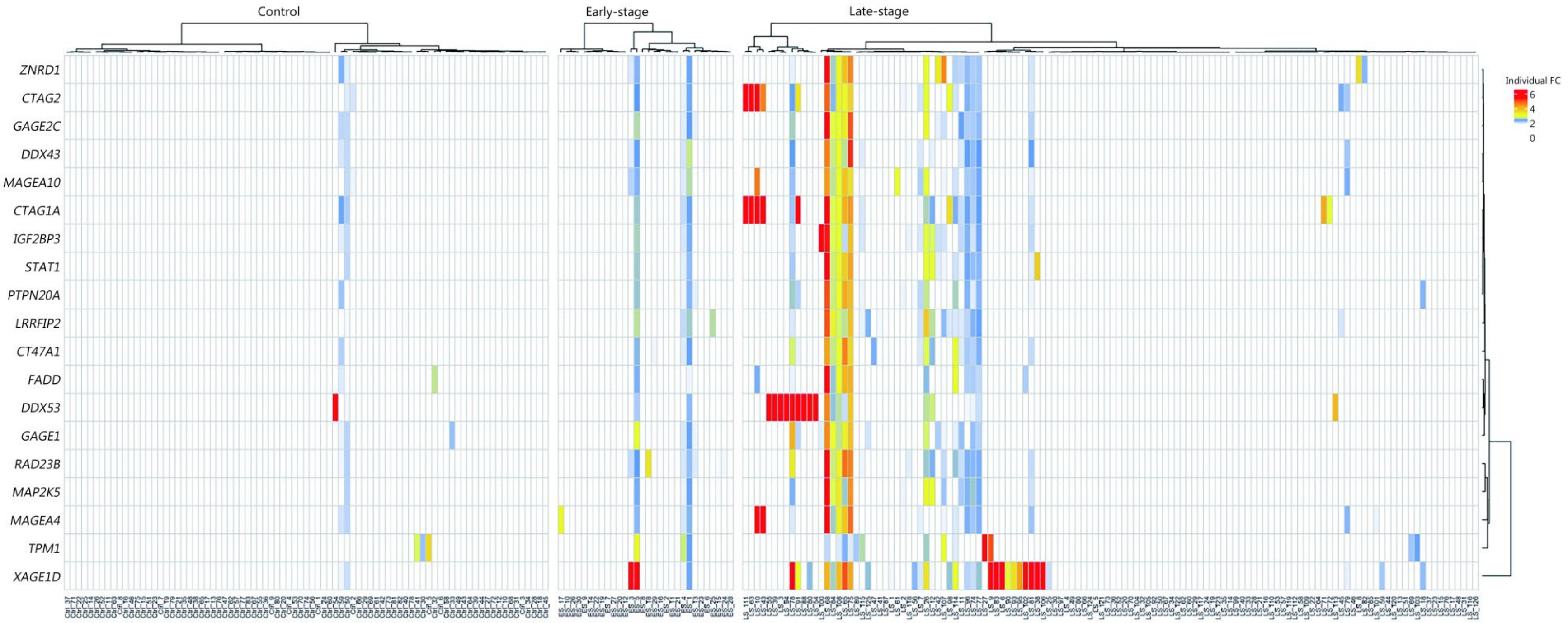

Fig. S2 Fold-change heatmap demonstrating autoantibody-based per-class sub-clusters. FC fold change, ZNRD1 RNA polymerase i subunit H, CTAG2 cancer/testis antigen 2, GAGE2C G antigen 2C, DDX43 DEAD-box helicase 43, MAGEA10 MAGE family member A10, CTAG1A cancer/testis antigen 1A, IGF2BP3 insulin like growth factor 2 MRNA binding protein 3, STAT1 signal transducer and activator of transcription 1, PTPN20A protein tyrosine phosphatase non-receptor type 20, LRRFIP2 LRR binding FLII interacting protein 2, CT47A1 cancer/testis antigen family 47 member A1, FADD Fas associated via death domain, DDX53 DEAD-box helicase 53, GAGE1 G antigen 1, RAD23B RAD23 homolog B, nucleotide excision repair protein, MAP2K5 mitogen-activated protein kinase kinase 5, MAGEA4 MAGE family member A4, TPM1 tropomyosin 1, XAGE1D X antigen family, member 1D
